# AGAINST: Fertility preservation for women with ovarian endometriosis: it is time to adopt this as routine practice

**DOI:** 10.1111/1471-0528.17166

**Published:** 2022-05-25

**Authors:** Martin Hirsch, Christian Becker, Melanie Davies

**Affiliations:** ^1^ Oxford Endometriosis CaRe Centre, Nuffield Department of Women's & Reproductive Health University of Oxford Oxford UK; ^2^ Elizabeth Garrett Anderson Institute for Women's Health University College London London UK

Fertility preservation (FP) is an established and recognised intervention for those undergoing gonadotoxic treatments, principally for malignancy. The surgical treatment of endometrioma and the disease itself reduce ovarian reserve and has sparked debate on whether FP should be offered prior to treatment.^1^

The association between endometriosis and infertility is accepted but uncertain as to aetiology and pathophysiology. A direct causation between surgery for ovarian endometrioma and reduced ovarian reserve may not be as clear as previously considered. Histological analyses confirm cortical follicular density and percentage of atretic follicles are negatively impacted within ovaries containing endometriomas compared with unaffected ovaries.^2^ This suggests that damage to the ovarian reserve may be partially inherent to the condition rather than iatrogenic, with no high‐quality evidence to support or refute the role of surgery ahead of ovarian stimulation.^3^ Tests used to assess ovarian reserve such as anti‐mullerian hormone and antral follicle count are highly predictive of ovarian response during ovarian stimulation, with the cumulative live birth rate directly linked to oocyte yield in FP,^4^ but these tests do not predict future fertility, fecundity or spontaneous conception. This is important for a cohort of patients considering FP having never tried to conceive.

When considering tangible outcomes, important to patients, the live birth rate does not differ among those undergoing surgery and expectant management of ovarian endometrioma ahead of *in vitro* fertilisation with enhanced spontaneous conception for those undergoing surgery.^3^ The largest observational studies of over 400 patients with endometrioma undergoing FP concluded that oocyte yield is lower for those having undergone surgery for endometrioma; however, there were no statistical differences in cumulative live birth rate in operated, unoperated and controls.^4^

The indication for FP in women with ovarian endometriosis remains unclear, as the natural history of endometriosis is poorly understood. The use of hormonal secondary prevention following cytoreductive surgery is safe, effective and recommended for patients with symptomatic ovarian endometriosis.^1^ Fertility preservation will be unnecessary for many and particularly young patients with normal or high ovarian reserve. This pre‐emptive intervention may contribute to health‐related anxiety and influence future health decision‐making without the guarantee of a live birth.

Globally, the fertility sector is variably regulated, with many non‐evidenced based interventions offered. In the UK, the Human Fertilisation and Embryology Authority regulates oocyte storage, but understanding the optimal approach to FP is hindered by low‐quality efficacy data on ovarian tissue cryopreservation, limited to case reports,^5^ and no assessment of harm. In the UK, the National Institute of Health and Care Excellence recommend that services are not considered for implementation prior to a robust cost‐effectiveness analysis. It is evidently clear that both clinicians and regulatory bodies currently lack high‐quality evidence to endorse routine usage of fertility preservation among women with ovarian endometrioma.^6^

We strongly advise against implementing a further fertility intervention until robust, impartial, randomised controlled trial data, including cost effectiveness, and patient perspectives can enable prognostic modelling for clinical guideline development in a nationally funded healthcare system.

## CONFLICT OF INTEREST

MH: None. CB: Current chair of European Society of Human Reproduction and Embryology Guideline Development Group for Endometriosis; Recipient of research grants from Bayer Healthcare, MDNA Life Sciences, Roche Diagnostics; Consultancy for Myovant, IDMC Member for ObsEva. MD: Chair of British Fertility Society fertility preservation special interest group. Completed disclosure of interest forms are available to view online as supporting information.

## Supporting information


ICMJE

ICMJE

ICMJE

## Data Availability

Data sharing is not applicable to this article as no new data were created or analyzed in this study.
